# Supplementary material for: Ultra-massive fluid transfusion in adult liver transplant recipients: A single center observational study
Source: PLoS One. 2025 Jun 17;20(6):e0325829. doi: 10.1371/journal.pone.0325829 (PMC12173374; doi:10.1371/journal.pone.0325829)
Supplement: S1 Table — (DOCX) [file pone.0325829.s001.docx]

**Supplementary Table 1.** Missing preoperative and intraoperative laboratory results across stages of liver transplant operation.

| **Variables (n = 81)** | **Missing number** |
| --- | --- |
| **Preoperative laboratory result** |  |
| Bilirubin | 27 (33.3%) |
| Fibrinogen | 7 (8.6%) |
| **Baseline laboratory results (Start of operation)** |  |
| Calcium | 7 (8.6%) |
| Glucose | 11 (13.6%) |
| Prothrombin Time | 5 (6.2%) |
| Platelet count | 5 (6.2%) |
| Fibrinogen | 7 (8.6%) |
| D-dimers | 11 (13.6%) |
| **Laboratory Results (Stage I of operation)** |  |
| Prothrombin Time | 7 (8.6%) |
| International Normalized Ratio | 6 (7.4%) |
| Activated Partial Thromboplastin Time | 6 (7.4%) |
| Platelet count | 6 (7.4%) |
| Fibrinogen | 6 (7.4%) |
| D-dimers | 6 (7.4%) |
| **Laboratory Results (Stage II of operation)** |  |
| Calcium | 9 (11.1%) |
| Glucose | 8 (9.9%) |
| Prothrombin Time | 5 (6.2%) |
| Platelet count | 5 (6.2%) |
| D-dimers | 5 (6.2%) |
| **Laboratory Results (Stage III of operation)** |  |
| Calcium | 7 (8.6%) |
| Glucose | 6 (7.4%) |
| **Laboratory Results (Closure of operation)** |  |
| Fibrinogen | 7 (8.6%) |
| D-dimers | 11 (13.6%) |
| Bicarbonate | 8 (9.9%) |
| Base excess | 6 (7.4%) |
| Chloride | 5 (6.2%) |
| Calcium | 8 (9.9%) |
| Glucose | 9 (11.1%) |
| Lactate | 5 (6.2%) |

Data are expressed as number and percentage of patients.
