# Supplementary material for: Ultra-massive fluid transfusion in adult liver transplant recipients: A single center observational study
Source: PLoS One. 2025 Jun 17;20(6):e0325829. doi: 10.1371/journal.pone.0325829 (PMC12173374; doi:10.1371/journal.pone.0325829)
Supplement: S2 Table — (DOCX) [file pone.0325829.s002.docx]

**Supplementary Table 2.** Preoperative and intraoperative laboratory results.

| **Variable (n = 81)** | **Measurement** |
| --- | --- |
| **Preoperative laboratory result** |  |
| INR | 1.6 (1.3‒2.1) |
| PT (s) | 18.8 (14.9‒27.0) |
| aPTT (s) | 41.0 (33.0‒57.0) |
| Fibrinogen (g/L) | 1.7 (1.2‒2.9) |
| Albumin (g/L) | 30.9 ± 6.6 |
| Platelet count (x 10^3^/μL) | 106.0 ± 71.0 |
| Hemoglobin (g/L) | 98.0 ± 20.4 |
| Creatinine (mg/L) | 115.2 ± 61.8 |
| Bilirubin (mg/L) | 251.2 ± 273.9 |
| Sodium (mmol/L) | 134.9 ± 5.9 |
| **Baseline coagulation profile: Start of operation** |  |
| INR | 19.0 (15.0‒28.3) |
| PT (s) | 1.7 (1.4‒2.4) |
| aPTT (s) | 41.0 (33.0‒57.0) |
| Platelet count (x 103/μL) | 93.2 ± 64.3 |
| Fibrinogen (g/L) | 1.7 (1.2‒2.9) |
| D-dimer (ng/mL) | 1,910.0 (1.8‒4,170.8) |
| **Coagulation profile: Stage I of operation** |  |
| INR | 21.5 (17.3‒29.2) |
| PT (s) | 1.9 (1.6‒2.5) |
| aPTT (s) | 46.0 (35.5‒64.5) |
| Platelet count (x 103/μL) | 90.4 ± 49.2 |
| Fibrinogen (g/L) | 1.4 (1.1‒2.0) |
| D-dimer (ng/mL) | 1,867.0 (1.6‒4,444.0) |
| **Coagulation profile: Stage II of operation** |  |
| INR | 48.8 (29.8‒90.5) |
| PT (s) | 4.4 (2.7‒8.7) |
| aPTT (s) | 165.0 (80.0‒201.0) |
| Platelet count (x 103/μL) | 45.0 ± 30.8 |
| Fibrinogen (g/L) | 0.7 (0.5‒0.8) |
| D-dimer (ng/mL) | 3,576.5 (3.6‒9,410.3) |
| **Coagulation profile: Stage III of operation** |  |
| INR | 75.0 (39.9‒121.0) |
| PT (s) | 6.4 (3.5‒11.0) |
| aPTT (s) | 201.0 (132.0‒201.0) |
| Platelet count (x 103/μL) | 45.0 ± 26.9 |
| Fibrinogen (g/L) | 0.7 (0.4‒0.8) |
| D-dimer (ng/mL) | 5,605.0 (5.9‒11,645.0) |
| **Coagulation profile: Closure of operation** |  |
| INR | 28.6 (24.7‒36.0) |
| PT (s) | 2.5 (2.1‒3.2) |
| aPTT (s) | 72.0 (56.0‒107.0) |
| Platelet count (x 103/μL) | 60.9 ± 27.5 |
| Fibrinogen (g/L) | 1.0 (0.8‒1.3) |
| D-dimer (ng/mL) | 4,748.5 (3.7‒7,566.0) |
| **Other laboratory result: Start of operation** |  |
| FiO_2_ (%) | 60.0 (50.0‒65.0) |
| pH | 7.34 ± 0.1 |
| pO_2_ (mmHg) | 218.1 ± 106.1 |
| pCO_2_ (mmHg) | 42.2 ± 6.2 |
| Bicarbonate (mmol/L) | 23.0 ± 4.7 |
| Sodium (mmol/L) | 134.9 ± 5.9 |
| Potassium (mmol/L) | 4.2 ± 0.7 |
| Chloride (mmol/L) | 104.4 ± 16.6 |
| Calcium (mmol/L) | 1.2 ± 0.3 |
| Hemoglobin (g/L) | 91.8 ± 20.5 |
| Glucose (mmol/L) | 7.3 ± 6.9 |
| Lactate (mmol/L) | 1.6 ± 1.2 |
| **Other laboratory result: Stage I of operation** |  |
| FiO_2_ (%) | 60.0 (50.0‒60.0) |
| pH | 7.3 ± 0.1 |
| pO_2_ (mmHg) | 241.6 ± 77.1 |
| pCO_2_ (mmHg) | 41.5 ± 44.3 |
| Bicarbonate (mmol/L) | 19.7 ± 4.6 |
| Sodium (mmol/L) | 134.8 ± 4.0 |
| Potassium (mmol/L) | 4.7 ± 4.1 |
| Chloride (mmol/L) | 100.4 ± 11.7 |
| Calcium (mmol/L) | 1.5 ± 4.1 |
| Hemoglobin (g/L) | 85.6 ± 19.1 |
| Glucose (mmol/L) | 5.8 ± 1.9 |
| Lactate (mmol/L) | 3.8 ± 2.0 |
| **Other laboratory result: Stage II of operation** |  |
| FiO_2_ (%) | 60.0 (50.0‒60.0) |
| pH | 7.3 ± 0.1 |
| pO_2_ (mmHg) | 220.1 ± 89.0 |
| pCO_2_ (mmHg) | 41.1 ± 5.7 |
| Bicarbonate (mmol/L) | 19.7 ± 3.7 |
| Sodium (mmol/L) | 136.8 ± 11.9 |
| Potassium (mmol/L) | 4.1 ± 0.6 |
| Chloride (mmol/L) | 99.8 ± 11.0 |
| Calcium (mmol/L) | 1.1 ± 0.2 |
| Hemoglobin (g/L) | 83.0 ± 15.3 |
| Glucose (mmol/L) | 9.4 ± 2.4 |
| Lactate (mmol/L) | 5.3 ± 2.4 |
| **Other laboratory result: Stage III of operation** |  |
| FiO_2_ (%) | 56.0 (50.0‒60.0) |
| pH | 7.3 ± 0.5 |
| pO_2_ (mmHg) | 203.9 ± 75.3 |
| pCO_2_ (mmHg) | 41.0 ± 7.6 |
| Bicarbonate (mmol/L) | 21.9 ± 4.0 |
| Sodium (mmol/L) | 136.6 ± 4.2 |
| Potassium (mmol/L) | 4.8 ± 4.4 |
| Chloride (mmol/L) | 99.8 ± 12.0 |
| Calcium (mmol/L) | 2.6 ± 12.3 |
| Hemoglobin (g/L) | 91.8 ± 19.1 |
| Glucose (mmol/L) | 10.4 ± 2.3 |
| Lactate (mmol/L) | 4.8 ± 3.3 |

Data are expressed as number (%), mean ± SD or median (interquartile range).

Abbreviation: INR, International Normalized Ratio; PT, Prothrombin Time; aPTT, Activated Partial Thromboplastin Time; FiO₂, Fraction of Inspired Oxygen; pH, Potential of Hydrogen; pO₂, Partial Pressure of Oxygen; pCO₂, Partial Pressure of Carbon Dioxide.
