# Supplementary material for: Ultra-massive fluid transfusion in adult liver transplant recipients: A single center observational study
Source: PLoS One. 2025 Jun 17;20(6):e0325829. doi: 10.1371/journal.pone.0325829 (PMC12173374; doi:10.1371/journal.pone.0325829)
Supplement: S3 Table — (DOCX) [file pone.0325829.s003.docx]

**Supplementary Table 3.** Postoperative complications by Clavien-Dindo classification of surgical complications in liver transplantation patients.

| **Complications** | **CD I** | **CD II** | **CD IIIa** | **CD IIIb** | **CD IVa** | **CD IVb** | **CD V** |
| --- | --- | --- | --- | --- | --- | --- | --- |
| **Surgical-specific** |  |  |  |  |  |  |  |
| Bleeding | 0 | 2 (2.5%) | 0 | 10 (12.3%) | 0 | 0 | 0 |
| Bile leak | 0 | 1 (1.2%) | 0 | 4 (4.9%) | 0 | 0 | 0 |
| Hepatic artery/vein thrombosis | 0 | 1 (1.2%) | 0 | 4 (4.9%) | 0 | 0 | 0 |
| Liver abscess | 0 | 1 (1.2%) | 0 | 0 | 0 | 0 | 0 |
| Other^1^ | 0 | 5 (6.2%) | 1 (1.2%) | 8 (9.9) | 0 | 0 | 0 |
| **Graft dysfunction** |  |  |  |  |  |  |  |
| Graft non-function^2^ | 0 | 0 | 0 | 7 (8.6%) | 0 | 0 | 0 |
| Long-term failure^3^ | 0 | 0 | 0 | 0 | 0 | 0 | 0 |
| **Cardiovascular** |  |  |  |  |  |  |  |
| Hypotension | 0 | 0 | 0 | 0 | 0 | 0 | 0 |
| Arrhythmia | 0 | 6 (7.4%) | 0 | 0 | 0 | 0 | 0 |
| Myocardial infarction | 0 | 1 (1.2%) | 0 | 0 | 0 | 0 | 0 |
| Syncope | 0 | 0 | 0 | 0 | 0 | 0 | 0 |
| Pericardial effusion | 0 | 0 | 0 | 2 (2.5%) | 0 | 0 | 0 |
| Other | 0 | 3 (3.7%) | 0 | 0 | 0 | 0 | 0 |
| **Pulmonary** |  |  |  |  |  |  |  |
| Pleural effusion | 0 | 10 (12.3%) | 0 | 0 | 0 | 0 | 0 |
| Atelectasis | 0 | 1 (1.2%) | 0 | 0 | 0 | 0 | 0 |
| Pneumonia | 0 | 11 (13.6%) | 0 | 0 | 0 | 0 | 0 |
| Pulmonary embolism | 0 | 2 (2.5%) | 0 | 0 | 0 | 0 | 0 |
| Respiratory failure | 0 | 2 (2.5%) | 0 | 0 | 0 | 0 | 0 |
| Other | 0 | 4 (4.9%) | 0 | 4 (4.9%) | 0 | 0 | 0 |
| **Hematology** |  |  |  |  |  |  |  |
| Anemia | 0 | 4 (4.9%) | 0 | 0 | 0 | 0 | 0 |
| Bacteremia | 10 (12.3%) | 0 | 0 | 0 | 0 | 0 | 0 |
| Other | 0 | 0 | 0 | 0 | 0 | 0 | 0 |
| **Gastrointestinal** |  |  |  |  |  |  |  |
| Ileus | 0 | 10 (12.3%) | 0 | 0 | 0 | 0 | 0 |
| Gastrointestinal bleeding | 0 | 0 | 0 | 1 (1.2%) | 0 | 0 | 0 |
| Ascites | 0 | 0 | 0 | 0 | 0 | 0 | 0 |
| Cholestasis | 0 | 0 | 0 | 0 | 0 | 0 | 0 |
| Other | 0 | 5 (6.2%) | 0 | 0 | 0 | 0 | 0 |
| **Renal** |  |  |  |  |  |  |  |
| Acute kidney injury | 0 | 9 (11.1%) | 0 | 0 | 0 | 0 | 0 |
| Urinary tract infection | 0 | 3 (3.7%) | 0 | 0 | 0 | 0 | 0 |
| Other | 0 | 0 | 0 | 0 | 0 | 0 | 0 |
| **Metabolic** |  |  |  |  |  |  |  |
| Electrolyte imbalance | 0 | 0 | 0 | 0 | 0 | 0 | 0 |
| Dysglycemia | 0 | 12 (14.8%) | 0 | 0 | 0 | 0 | 0 |
| Other | 0 | 3 (3.7%) | 0 | 0 | 0 | 0 | 0 |
| **Neurologic** |  |  |  |  |  |  |  |
| Stroke | 0 | 0 | 0 | 0 | 0 | 0 | 0 |
| Other | 0 | 1 (1.2%) | 0 | 0 | 0 | 0 | 0 |
| **Infection** |  |  |  |  |  |  |  |
| Sepsis | 0 | 11 (13.6%) | 0 | 0 | 1 (1.2%) | 1 (1.2%) | 2 (2.4%) |
| Intra-abdominal collection | 0 | 0 | 0 | 1 (1.2%) | 0 | 0 | 0 |
| Other | 0 | 0 | 0 | 0 |  | 0 | 0 |

Data are expressed as number and percentage of patients who developed a postoperative complication.
^1^Other complications include bowel perforation; gastric perforation; anastomotic biliary stricture; bile leak; biloma; cholangitis; esophageal perforation; perihepatic collection; portal vein stenosis; hepatic collections; sub-hepatic collection; hepatic artery dissection.
^2^Primary graft non-function/early allograft dysfunction.
^3^Graft loss beyond 30 days post-transplant.

Abbreviation: CD, Clavien-Dindo classification of surgical complications.
