# Supplementary material for: Ultra-massive fluid transfusion in adult liver transplant recipients: A single center observational study
Source: PLoS One. 2025 Jun 17;20(6):e0325829. doi: 10.1371/journal.pone.0325829 (PMC12173374; doi:10.1371/journal.pone.0325829)
Supplement: S5 Table — (DOCX) [file pone.0325829.s005.docx]

**Supplementary Table 5.** Associations between intraoperative and postoperative fluid/transfusion volumes and postoperative complications, including resuscitation shift.

| **Postoperative complication** | **Variable** | **Time point** | **OR (95%CI)** | **P value** |
| --- | --- | --- | --- | --- |
| Surgery-specific complications | Total volume (mL) | Intraoperative | 0.98 (0.92 – 1.03) | 0.574 |
|  |  | Postoperative 24hr | 0.60 (0.31 – 1.14) | 0.125 |
|  |  | Resuscitation shift | 1.02 (0.96 – 1.08) | 0.448 |
|  | RBC transfusion (units) | Intraoperative | 1.13 (0.91 – 1.41) | 0.268 |
|  |  | Postoperative 24hr | 0.35 (0.13 – 0.97) | 0.043* |
|  |  | Resuscitation shift | 0.95 (0.83 – 1.09) | 0.460 |
|  | Transfusion volume (100mL) | Intraoperative | 100.0 (99.97 – 100.06) | 0.735 |
|  |  | Postoperative 24hr | 99.91 (99.80 – 100.01) | 0.106 |
|  |  | Resuscitation shift | 99.99 (99.97 – 100.01) | 0.589 |
| Surgery specific complications: Bleeding | Total volume (mL) | Intraoperative | - | - |
|  |  | Postoperative 24hr | - | - |
|  |  | Resuscitation shift | - | - |
|  | RBC transfusion (units) | Intraoperative | - | - |
|  |  | Postoperative 24hr | - | - |
|  |  | Resuscitation shift | - | - |
|  | Transfusion volume (100mL) | Intraoperative | - | - |
|  |  | Postoperative 24hr | - | - |
|  |  | Resuscitation shift | - | - |
| No. of complications ≥ 3 | Total volume (mL) | Intraoperative | 1.01 (0.96 ‒ 1.06) | 0.801 |
|  |  | Postoperative 24hr | 1.00 (0.63 ‒ 1.57) | 0.986 |
|  |  | Resuscitation shift | 0.99 (0.94 ‒ 1.05) | 0.802 |
|  | RBC transfusion (units) | Intraoperative | 0.93 (0.60 ‒ 1.43) | 0.726 |
|  |  | Postoperative 24hr | 1.07 (0.91 ‒ 1.26) | 0.372 |
|  |  | Resuscitation shift | 0.94 (0.83 ‒ 1.07) | 0.348 |
|  | Transfusion volume (100mL) | Intraoperative | 100.01 (99.98 – 100.04) | 0.406 |
|  |  | Postoperative 24hr | 100.00 (99.94 – 100.06) | 0.997 |
|  |  | Resuscitation shift | 99.99 (99.97 – 100.02) | 0.450 |
| CDC grade ≥ 3 | Total volume (mL) | Intraoperative | 1.01 (0.95 ‒ 1.07) | 0.659 |
|  |  | Postoperative 24hr | 1.49 (0.96 ‒ 2.33) | 0.074 |
|  |  | Resuscitation shift | 0.99 (0.94 ‒ 1.05) | 0.748 |
|  | RBC transfusion (units) | Intraoperative | 1.25 (0.93 ‒ 1.70) | 0.126 |
|  |  | Postoperative 24hr | 1.49 (1.00-2.22) | 0.048* |
|  |  | Resuscitation shift | 0.92 (0.80 ‒ 1.06) | 0.298 |
|  | Transfusion volume (100mL) | Intraoperative | 100.02 (99.98 – 100.06) | 0.2142 |
|  |  | Postoperative 24hr | 100.05 (99.99 – 100.11) | 0.088 |
|  |  | Resuscitation shift | 99.99 (99.96 – 100.02) | 0.514 |
| Return to theatre: Bleeding | Total volume (mL) | Intraoperative | - | - |
|  |  | Postoperative 24hr | - | - |
|  |  | Resuscitation shift | - | - |
|  | RBC transfusion (units) | Intraoperative | - | - |
|  |  | Postoperative 24hr | - | - |
|  |  | Resuscitation shift | - | - |
|  | Transfusion volume (100mL) | Intraoperative | 99.93 (99.85 – 100.02) | 0.132 |
|  |  | Postoperative 24hr | 100.07 (99.94 – 100.19) | 0.292 |
|  |  | Resuscitation shift | 100.07 (99.99 – 100.15) | 0.119 |
| 30-day mortality | Total volume (mL) | Intraoperative | - | - |
|  |  | Postoperative 24hr | - | - |
|  |  | Resuscitation shift | - | - |
|  | RBC transfusion (units) | Intraoperative | - | - |
|  |  | Postoperative 24hr | - | - |
|  |  | Resuscitation shift | - | - |
|  | Transfusion volume (100mL) | Intraoperative | - | - |
|  |  | Postoperative 24hr | - | - |
|  |  | Resuscitation shift | - | - |

The term resuscitation shift was operationally defined as the net change in postoperative fluid or transfusion volume administered compared to the volume of fluid administration intraoperatively. Two logistic regression models were adjusted for perioperative covariates to analyze these relationships. Models for intraoperative and postoperative variables of fluid or transfusion were fitted simultaneously to assess their independent effects. As a supplementary sensitivity analysis, the resuscitation shift model (termed the trajectory model) quantified the net volume difference between intraoperative and postoperative 24-hour periods. –: denotes model convergence failure or insufficient data for reliable estimation. These models were excluded from interpretation due to instability or low event rates. *: P<0.05.
