# Supplementary material for: Ultra-massive fluid transfusion in adult liver transplant recipients: A single center observational study
Source: PLoS One. 2025 Jun 17;20(6):e0325829. doi: 10.1371/journal.pone.0325829 (PMC12173374; doi:10.1371/journal.pone.0325829)
Supplement: S6 Table — (DOCX) [file pone.0325829.s006.docx]

**Supplementary Table 6.** Adjusted relationship between intraoperative and first 24-hour postoperative PRBC transfusion and postoperative complications.

| **PRBCs transfusion (unit)** | **Intraoperative** | | **Postoperative** | | **Overall** | |
| --- | --- | --- | --- | --- | --- | --- |
|  | **OR (95% CI)** | **p-value** | **OR (95% CI)** | **p-value** | **OR (95% CI)** | **p-value** |
| **Presence of any complication** | 0.25 (0 – inf) | >0.99 | 118.67 (0 – inf) | >0.99 | 0.32 (0 – inf) | >0.99 |
| **Surgery-specific complication** | | | | | | |
| Bleeding | 16.29 (0 – inf) | >0.99 | 8.93 (0 – inf) | >0.99 | 7.98 (0 – inf) | >0.99 |
| Bile leakage | 5.95 (0 – inf) | >0.99 | 573.37 (0 – inf) | >0.99 | 4.71 (0 – inf) | >0.99 |
| Hepatic artery/vein thrombosis | 0.18 (0 – inf) | >0.99 | 0 (0 – inf) | >0.99 | 0.15 (0 – inf) | >0.99 |
| Liver abscess | 1.01 (0 – inf) | >0.99 | 0.45 (0 – inf) | >0.99 | 1 (0 – inf) | >0.99 |
| Others | 0 (0 – inf) | >0.99 | 0 (0 – inf) | >0.99 | 0 (0 – inf) | >0.99 |
| **Graft function** | | | | | | |
| Graft non-function¹ | 4.74 (0 – inf) | >0.99 | 87.65 (0 – inf) | >0.99 | 5.06 (0 – inf) | >0.99 |
| Long-term failure² | 1 (0 – inf) | >0.99 | 1 (0 – inf) | >0.99 | 1 (0 – inf) | >0.99 |
| **Reoperation/interventions** | | | | | | |
| All-cause | 181.46 (0 – inf) | >0.99 | 0 (0 – inf) | >0.99 | 721.55 (0 – inf) | >0.99 |
| Infection | 2851.93 (0 – inf) | >0.99 | 0.08 (0 – inf) | >0.99 | 116.04 (0 – inf) | >0.99 |
| **Mortality** | | | | | | |
| On-table mortality | 6.16 (0 – inf) | >0.99 | 1 (0 – inf) | >0.99 | 1.18 (0 – inf) | >0.99 |
| In-hospital mortality | 1 (0 – inf) | >0.99 | 1 (0 – inf) | >0.99 | 1 (0 – inf) | >0.99 |
| 30-day mortality | 3.22 (0 – inf) | >0.99 | 2.94 (0 – inf) | >0.99 | 1.23 (0 – inf) | >0.99 |
| 1-year mortality | 19.67 (0 – inf) | >0.99 | 0.6 (0 – inf) | >0.99 | 13.84 (0 – inf) | >0.99 |
| 5-year mortality | 9.74×10⁶ (0 – inf) | >0.99 | 1.46×10⁶ (0 – inf) | >0.99 | 4.04×10⁵ (0 – inf) | >0.99 |
| Overall mortality | 1.81×10⁶ (0 – inf) | >0.99 | 1.78×10⁶ (0 – inf) | >0.99 | 1.25×10⁶ (0 – inf) | >0.99 |

Data are presented as odds ratios (ORs) with 95% confidence intervals (CIs) from logistic regression for complication outcomes, along with corresponding p-values. Statistical significance is indicated by * (p < 0.05).
^1^ Primary graft non-function/early allograft dysfunction.
^2^ Graft loss beyond 30 days post-transplant. PRBCs: packed red blood cells.
