# Supplementary material for: Ultra-massive fluid transfusion in adult liver transplant recipients: A single center observational study
Source: PLoS One. 2025 Jun 17;20(6):e0325829. doi: 10.1371/journal.pone.0325829 (PMC12173374; doi:10.1371/journal.pone.0325829)
Supplement: S7 Table — (DOCX) [file pone.0325829.s007.docx]

**Supplementary Table 7.** Adjusted relationship between intraoperative and first 24-hour postoperative resuscitation volume and postoperative complications.

| **Fluid and blood products (mL)** | **Intraoperative** | | **Postoperative** | | **Overall** | |
| --- | --- | --- | --- | --- | --- | --- |
|  | **OR (95% CI)** | **p-value** | **OR (95% CI)** | **p-value** | **OR (95% CI)** | **p-value** |
| **Presence of any complication** | 0.52 (0 – inf) | >0.99 | 794.87 (0 – inf) | >0.99 | 0.59 (0 – inf) | >0.99 |
| **Surgery-specific complication** | | | | | | |
| Bleeding | 2.12 (0 – inf) | >0.99 | 16.58 (0 – inf) | >0.99 | 2.01 (0 – inf) | >0.99 |
| Bile leakage | 1.83 (0 – inf) | >0.99 | 565.94 (0 – inf) | >0.99 | 1.78 (0 – inf) | >0.99 |
| Hepatic artery/vein thrombosis | 0.31 (0 – inf) | >0.99 | 0 (0 – inf) | >0.99 | 0.39 (0 – inf) | >0.99 |
| Liver abscess | 0.91 (0 – inf) | >0.99 | 0.26 (0 – inf) | >0.99 | 0.91 (0 – inf) | >0.99 |
| Others | 0.25 (0 – inf) | >0.99 | 0 (0 – inf) | >0.99 | 0.25 (0 – inf) | >0.99 |
| **Graft function** | | | | | | |
| Graft non-function¹ | 1.92 (0 – inf) | >0.99 | 109.11 (0 – inf) | >0.99 | 1.9 (0 – inf) | >0.99 |
| Long-term failure² | 1 (0 – inf) | >0.99 | 1 (0 – inf) | >0.99 | 1 (0 – inf) | >0.99 |
| **Reoperation/interventions** | | | | | | |
| All-cause | 5.91 (0 – inf) | >0.99 | 0.01 (0 – inf) | >0.99 | 6.95 (0 – inf) | >0.99 |
| Infection | 3.13 (0 – inf) | >0.99 | 2.55 (0 – inf) | >0.99 | 3.37 (0 – inf) | >0.99 |
| **Mortality** | | | | | | |
| On-table mortality | 1.49 (0 – inf) | >0.99 | 1 (0 – inf) | >0.99 | 1.15 (0 – inf) | >0.99 |
| In-hospital mortality | 1 (0 – inf) | >0.99 | 1 (0 – inf) | >0.99 | 1 (0 – inf) | >0.99 |
| 30-day mortality | 1.42 (0 – inf) | >0.99 | 5.42 (0 – inf) | >0.99 | 1.11 (0 – inf) | >0.99 |
| 1-year mortality | 2.09 (0 – inf) | >0.99 | 0.45 (0 – inf) | >0.99 | 2.24 (0 – inf) | >0.99 |
| 5-year mortality | 47.76 (0 – inf) | >0.99 | 2.63×10⁸ (0 – inf) | >0.99 | 146.29 (0 – inf) | >0.99 |
| Overall mortality | 19.51 (0 – inf) | >0.99 | 3.05×10⁷ (0 – inf) | >0.99 | 66.97 (0 – inf) | >0.99 |

Data are presented as odds ratios (ORs) with 95% confidence intervals (CIs) from logistic regression for complication outcomes, along with corresponding p-values. Statistical significance is indicated by * (p < 0.05).
^1^ Primary graft non-function/early allograft dysfunction.
^2^ Graft loss beyond 30 days post-transplant.
