# Supplementary material for: Ultra-massive fluid transfusion in adult liver transplant recipients: A single center observational study
Source: PLoS One. 2025 Jun 17;20(6):e0325829. doi: 10.1371/journal.pone.0325829 (PMC12173374; doi:10.1371/journal.pone.0325829)
Supplement: S8 Table — (DOCX) [file pone.0325829.s008.docx]

**Supplementary Table 8.** Impact of FFP: PRBC and platelet: PRBC ratios on recovery outcomes in liver transplantation patients.

|  | **PRBCs** |  | **FFP Ratio** |  | **Interaction** |  |
| --- | --- | --- | --- | --- | --- | --- |
| **Co-transfused FFP and PRBCs** | **Coefficient (95% CI)** | **p-value** | **Coefficient (95% CI)** | **p-value** | **Coefficient (95% CI)** | **p-value** |
| **Mechanical ventilation (hrs)** | -1.34 (–14.54 – 11.86) | 0.843 | -187.19 (–508.25 – 133.87) | 0.261 | 29.84 (10.25 – 49.44) | 0.005* |
| **ICU LOS** | 0.06 (–1.05 – 1.18) | 0.911 | -10.25 (–37.35 – 16.84) | 0.464 | 1.99 (0.34 – 3.65) | 0.024* |
| **Hospital LOS** | -0.14 (–1.97 – 1.69) | 0.882 | -25.17 (–69.57 – 19.22) | 0.274 | 4.01 (1.29 – 6.73) | 0.007* |
|  | **PRBCs** |  | **Platelet Ratio** |  | **Interaction** |  |
| **Co-transfused platelet and PRBCs** | **Coefficient (95% CI)** | **p-value** | **Coefficient (95% CI)** | **p-value** | **Coefficient (95% CI)** | **p-value** |
| **Mechanical ventilation (hrs)** | 8.66 (0.32 – 17.01) | 0.05 | -360.96 (–790.23 – 68.3) | 0.109 | 57.12 (27.66 – 86.58) | 0.001* |
| **ICU LOS** | 1.06 (0.36 – 1.76) | 0.005* | 5.51 (–30.31 – 41.34) | 0.765 | 3.18 (0.72 – 5.64) | 0.016* |
| **Hospital LOS** | 2.21 (0.87 – 3.55) | 0.003* | 8.37 (–61.71 – 78.45) | 0.816 | 1.65 (–3.04 – 6.34) | 0.495 |

Data are presented as correlation coefficients (r) with 95% confidence intervals (CIs) from linear regression for recovery outcomes, along with corresponding p-values. * Indicates statistical significance (p < 0.05). FFP, fresh frozen plasma; PRBCs: packed red blood cells; ICU: intensive care unit; LOS: length of stay.
