# Supplementary material for: Ultra-massive fluid transfusion in adult liver transplant recipients: A single center observational study
Source: PLoS One. 2025 Jun 17;20(6):e0325829. doi: 10.1371/journal.pone.0325829 (PMC12173374; doi:10.1371/journal.pone.0325829)
Supplement: S9 Table — (DOCX) [file pone.0325829.s009.docx]

**Supplementary Table 9.** Impact of FFP: PRBC ratios on complications in liver transplantation patients.

| **Co-transfused FFP and PRBCs** | **PRBCs** | | **FFP ratio** | | **Interaction** | |
| --- | --- | --- | --- | --- | --- | --- |
|  | **OR (95% CI)** | **p-value** | **OR (95% CI)** | **p-value** | **OR (95% CI)** | **p-value** |
| **Complications** | | | | | | |
| No. of complications (≥3) | 1.21 (0.87 ‒ 1.7) | 0.254 | 432.6 (0.06 ‒ 3.05×10^5^) | 0.179 | 0.78 (0.46 ‒ 1.31) | 0.341 |
| Severe complications (CVD ≥ 3) | 0 (0 ‒ inf) | >0.99 | 0 (0 ‒ inf) | >0.99 | 4.24×10^30^ (0 ‒ inf) | >0.99 |
| Presence of any complication | 0.04 (0 ‒ inf) | >0.99 | 0 (0 ‒ inf) | >0.99 | 307.12 (0 ‒ inf) | >0.99 |
| Presence of any surgical-specific complication | 0.97 (0.74 ‒ 1.28) | 0.844 | 0.24 (0 ‒ 390.31) | 0.708 | 1 (0.64 ‒ 1.56) | >0.99 |
| **Surgical-specific complication** | | | | | | |
| Bleeding | 1 (0 ‒ inf) | >0.99 | 1 (0 ‒ inf) | >0.99 | 1 (0 ‒ inf) | >0.99 |
| Bile leakage | 0.12 (0 ‒ inf) | >0.99 | 0 (0 ‒ inf) | >0.99 | 340.07 (0 ‒ inf) | >0.99 |
| Hepatic artery/vein thrombosis | 38.92 (0 ‒ inf) | >0.99 | 0 (0 ‒ inf) | >0.99 | 0.01 (0 ‒ inf) | >0.99 |
| Liver abscess | 2.49 (0 ‒ inf) | >0.99 | 1.66×10^12^ (0 ‒ inf) | >0.99 | 0.08 (0 ‒ inf) | >0.99 |
| Others | 0 (0 ‒ inf) | >0.99 | 0 (0 ‒ inf) | >0.99 | 2.37×10^8^ (0 ‒ inf) | >0.99 |
| **Graft function** | | | | | | |
| Graft non-function^1^ | 30.81 (0 ‒ inf) | >0.99 | 6.40×10^16^ (0 ‒ inf) | >0.99 | 0.06 (0 ‒ inf) | >0.99 |
| Long-term failure^2^ | 1.11 (0 ‒ inf) | >0.99 | 0 (0 ‒ inf) | >0.99 | 11.5 (0 ‒ inf) | >0.99 |

Data are presented as odds ratios (ORs) with 95% confidence intervals (CIs) from logistic regression for complication outcomes, along with corresponding p-values. Statistical significance is indicated by * (p < 0.05).
