# Supplementary material for: Ultra-massive fluid transfusion in adult liver transplant recipients: A single center observational study
Source: PLoS One. 2025 Jun 17;20(6):e0325829. doi: 10.1371/journal.pone.0325829 (PMC12173374; doi:10.1371/journal.pone.0325829)
Supplement: S10 Table — (DOCX) [file pone.0325829.s010.docx]

**Supplementary Table 10.** Impact of Platelet:PRBC ratios on complications in liver transplantation patients.

| **Co-transfused Platelet and PRBCs** | **PRBCs** | | **Platelet ratio** | | **Interaction** | |
| --- | --- | --- | --- | --- | --- | --- |
|  | **OR (95% CI)** | **p-value** | **OR (95% CI)** | **p-value** | **OR (95% CI)** | **p-value** |
| **Complications** | | | | | | |
| No. of complications (≥3) | 1.17 (0.89 ‒ 1.53) | 0.258 | 3.43×10^3^ (0 ‒ 4.60×10^9^) | 0.258 | 0.61 (0.22 ‒ 1.73) | 0.354 |
| Severe complications (CVD ≥ 3) | 1.24 (0.86 ‒ 1.79) | 0.239 | 0 (0 ‒ 7.7×10^4^) | 0.219 | 1.99 (0.51 ‒ 7.84) | 0.324 |
| Presence of any complication | 00.34 (0 ‒ inf) | >0.99 | 0 (0 ‒ inf) | >0.99 | 2.37 (0 ‒ inf) | >0.99 |
| Presence of any surgical-specific complication | 0.87 (0.72 ‒ 1.06) | 0.175 | 0 (0 ‒ 15.95) | 0.124 | 1.02 (0.47 ‒ 2.22) | 0.967 |
| **Surgical-specific complication** | | | | | | |
| Bleeding | 14.12 (0 ‒ inf) | >0.99 | 3.81×10^30^ (0 ‒ inf) | >0.99 | 0.02 (0 ‒ inf) | >0.99 |
| Bile leakage | 6.91 (0 ‒ inf) | >0.99 | 3.48×10^12^ (0 ‒ inf) | >0.99 | 0 (0 ‒ inf) | >0.99 |
| Hepatic artery/vein thrombosis | 0 (0 ‒ inf) | >0.99 | 0 (0 ‒ inf) | >0.99 | 1.77×10^13^ (0 ‒ inf) | >0.99 |
| Liver abscess | 0.66 (0 ‒ inf) | >0.99 | 0 (0 ‒ inf) | >0.99 | 0.69(0 ‒ inf) | >0.99 |
| Others | 0.28 (0 ‒ inf) | >0.99 | 7.00×10^70^ (0 ‒ inf) | >0.99 | 0 (0 ‒ inf) | >0.99 |
| **Graft function** | | | | | | |
| Graft non-function^1^ | 4.5 (0 ‒ inf) | >0.99 | 0 (0 ‒ inf) | >0.99 | 0.39 (0 ‒ inf) | >0.99 |
| Long-term failure^2^ | 1 (0 ‒ inf) | >0.99 | 1 (0 ‒ inf) | >0.99 | 1 (0 ‒ inf) | >0.99 |
| **Reoperation or interventions** | | | | | | |
| All-cause | 7.15 (0 ‒ inf) | >0.99 | 0 (0 ‒ inf) | >0.99 | 1.43×10^4^ (0 ‒ inf) | >0.99 |
| Bleeding | 0.77 (0.44 ‒ 1.36) | 0.367 | 0 (0 ‒ 1.85×10^15^) | 0.517 | 0.87 (0.03 ‒ 21.86) | 0.934 |
| Infection | 1 (0 ‒ inf) | >0.99 | 1 (0 ‒ inf) | >0.99 | 1 (0 ‒ inf) | >0.99 |

Data are presented as odds ratios (ORs) with 95% confidence intervals (CIs) from logistic regression for complication outcomes, along with corresponding p-values. Statistical significance is indicated by * (p < 0.05).
^1^ Primary graft non-function/early allograft dysfunction.
^2^ Graft loss beyond 30 days post-transplant. PRBCs: packed red blood cells.
