# Supplementary material for: Ultra-massive fluid transfusion in adult liver transplant recipients: A single center observational study
Source: PLoS One. 2025 Jun 17;20(6):e0325829. doi: 10.1371/journal.pone.0325829 (PMC12173374; doi:10.1371/journal.pone.0325829)
Supplement: S11 Table — (DOCX) [file pone.0325829.s011.docx]

**Supplementary Table 11.** Impact of Cryoprecipitate: PRBC ratios on complications in liver transplantation patients.

| **Co-transfused cryoprecipitate and PRBCs** | **PRBCs** | | **Cryoprecipitate ratio** | | | **Interaction** | | |
| --- | --- | --- | --- | --- | --- | --- | --- | --- |
|  | **OR (95% CI)** | **p-value** | **OR (95% CI)** | **p-value** | | **OR (95% CI)** | | **p-value** |
| **Complications** | | | | | | | | |
| No. of complications (≥3) | 1.21 (0.88 ‒ 1.67) | 0.229 | 4.12 (0.45 ‒ 37.29) | 0.208 | | 0.93 (0.79 ‒ 1.09) | | 0.36 |
| Severe complications (CVD ≥ 3) | 1.13 (0.71 ‒ 1.78) | 0.609 | 0.28 (0 ‒ 20.67) | 0.559 | | 1.08 (0.87 ‒ 1.35) | | 0.469 |
| Presence of any complication | 0.14 (0 ‒ inf) | >0.99 | 0 (0 ‒ inf) | >0.99 | | 2.49 (0 ‒ inf) | | >0.99 |
| Presence of any surgical-specific complication | 0 (0 ‒ inf) | >0.99 | 0 (0 ‒ inf) | >0.99 | | 4015931.97 (0 ‒ inf) | | >0.99 |
| **Surgical-specific complication** | | | | | | | | |
| Bleeding | 21.88 (0 ‒ inf) | >0.99 | 3131021.92 (0 ‒ inf) | >0.99 | | 0.43 (0 ‒ inf) | | >0.99 |
| Bile leakage | 1.2 (0 ‒ inf) | >0.99 | 6723.47 (0 ‒ inf) | >0.99 | | 1.61 (0 ‒ inf) | | >0.99 |
| Hepatic artery/vein thrombosis | 0.01 (0 ‒ inf) | >0.99 | 0 (0 ‒ inf) | >0.99 | | 4.47 (0 ‒ inf) | | >0.99 |
| Liver abscess | 0.74 (0 ‒ inf) | >0.99 | 0.06 (0 ‒ inf) | >0.99 | | 1.23 (0 ‒ inf) | | >0.99 |
| Others | 0 (0 ‒ inf) | >0.99 | 0 (0 ‒ inf) | >0.99 | | 4015931.97 (0 ‒ inf) | | >0.99 |
| **Graft function** | | | | | | | | |
| Graft non-function^1^ | 2.36 (0 ‒ inf) | >0.99 | 0 (0 ‒ inf) | | >0.99 | | 1.66 (0 ‒ inf) | >0.99 |
| Long-term failure^2^ | 1 (0 ‒ inf) | >0.99 | 1 (0 ‒ inf) | | >0.99 | | 1 (0 ‒ inf) | >0.99 |
| **Reoperation or interventions** | | | | | | | | |
| All-cause | 11017.17 (0 ‒ inf) | >0.99 | 89534512283.13 (0 ‒ inf) | | >0.99 | | 0.15 (0 ‒ inf) | >0.99 |
| Bleeding | e^1.12*10^13^ (Inf ‒ Inf) | <0.001 | e^4.11*10^14^ (Inf ‒ Inf) | | <0.001 | | e^-4.69*10^13^ (0 ‒ 0) | <0.001 |
| Infection | 1 (0 ‒ inf) | >0.99 | 1 (0 ‒ inf) | | >0.99 | | 1 (0 ‒ inf) | >0.99 |

Data are presented as odds ratios (ORs) with 95% confidence intervals (CIs) from logistic regression for complication outcomes, along with corresponding p-values. Statistical significance is indicated by * (p < 0.05).
^1^ Primary graft non-function/early allograft dysfunction.
^2^ Graft loss beyond 30 days post-transplant. PRBCs: packed red blood cells.
