# Supplementary material for: Ultra-massive fluid transfusion in adult liver transplant recipients: A single center observational study
Source: PLoS One. 2025 Jun 17;20(6):e0325829. doi: 10.1371/journal.pone.0325829 (PMC12173374; doi:10.1371/journal.pone.0325829)
Supplement: Attachment — Submitted filename: RESPONSE to REVIEWERS_20April2025.pdf [file pone.0325829.s013.pdf]

Ryan M. Thomas, M.D
Academic EditorPLoS ONE

Ref: Resubmission of PONE-D-25-06585

Title: Ultra-massive fluid transfusion in adult liver transplant recipients: a single center observational study

Dear Dr. Thomas:

My coauthors and I would like to thank you and the expert reviewers for taking the time to provide a very constructive and thoughtful review of our manuscript.

As requested, we have included the following items with our resubmission:

1. A point-by-point response letter (to follow) that addresses each issue raised by the Academic Editor and the Reviewers. I have uploaded this letter as a separate file labelled "Response to Reviewers."
2. A marked-up copy of the manuscript that highlights changes made to the original version. This has been uploaded as a separate file labelled "Revised Manuscript with Tracked Changes."
3. An unmarked version of our revised paper without tracked changes. This has also been uploaded as a separate file labelled "Manuscript."

Review of Comments to the Author

We thank the reviewer for their expert insights and time spent reviewing our manuscript. We have provided a detailed comment to each of the reviewer's questions, as outlined below.

1. **Reviewer 1, comment 1:** A significant limitation of the study is the lack of analysis on predictors of massive transfusion. Given the challenges in managing patients requiring ultra-massive transfusions, it would be beneficial to identify predictors of massive transfusion and the volume of transfusion required. This could include previously identified predictors such as those reported by Rana et al. (Ref 23), which include previous abdominal operations, warm ischemia time, hepatectomy time, and bilirubin levels.

Authors' response to R1 comment 1: Thank you for your feedback on the importance of predictors of massive transfusion.

We acknowledge the importance of identifying predictors of massive transfusion and transfusion volume, given the complexity inherent in managing these patients. However, the design of our study differs substantially from that of Rana et al. In their retrospective analysis of 233 consecutive liver transplant recipients, Rana and colleagues included both patients who did and did not receive a massive transfusion. Through this approach, they identified four significant risk factors for intraoperative blood requirements when comparing patients who required transfusion to those who did not: warm ischemia time (OR 1.12, 95% CI 1.06–1.18), bilirubin (OR 1.04, 95% CI 1.02–1.08), previous surgery (OR 1.7, 95% CI 1.02–2.9), and hepatectomy time (OR 1.01, 95% CI 1.00–1.02). Specifically, warm ischemia time, bilirubin, prior surgery, and hepatectomy time were 12%, 4%, 70%, and 1% higher, respectively, in the transfused group compared to the non-transfused group.

In our study we are unable to explore predictors of ultra-massive fluid transfusion (UMFT). The development of a valid prediction model for UMFT in our study requires the inclusion of both patients who received UMFT and those who did not. The absence of a "control group" precludes meaningful statistical comparison of predictor variables, thereby impeding any model's ability to distinguish factors truly associated with UMFT from random variation. Given that our study only included cases of UMFT,

this “imbalance” increases the risk of overfitting and undermines the reliability of the model in this liver transplant population. This has been briefly updated in the limitations section.

We appreciate the author’s insightful comment and agree that this represents an excellent direction for future research, which we are now actively exploring.

2. **Reviewer 1, comment 2:** The study does not provide details on the timing of fluid administration during the perioperative period. Differentiating when patients received fluids—such as up to hepatectomy, post-reperfusion, and postoperatively—would offer insights into specific phases of surgery where fluid management is most critical. Detailed reporting on this aspect would enhance understanding of fluid administration's impact, as well as its causes, at various surgical stages. A single center study with access to granular data should have this as its major advantage over a larger multicenter or national database study

Authors’ response to R1 comment 2: Thank you for this insightful suggestion. We concur that the timing of fluid administration is both informative and clinically significant. In response, we have conducted a comprehensive review of each medical record to accurately determine the timing of all fluids administered. This information is now presented as an additional figure (Figure 2), which graphically depicts the volume and type of fluids and blood products administered at each stage of liver transplantation—including induction of anesthesia, liver dissection phase, anhepatic phase, reperfusion, and neohepatic phase—as well as during the first 12 hours and the 12–24 hour period postoperatively.

The data clearly demonstrate that most of the fluid administration occurs intraoperatively, particularly during the liver dissection phase representing a period of particularly high fluid and blood product usage, consistent with its known association with increased bleeding risk.

We believe that this figure provides a comprehensive and visually accessible representation of perioperative fluid and blood product utilization, effectively illustrating patterns of administration across the various phases of liver transplantation and the immediate postoperative period.

We have uploaded **Fig 2** as a new file.

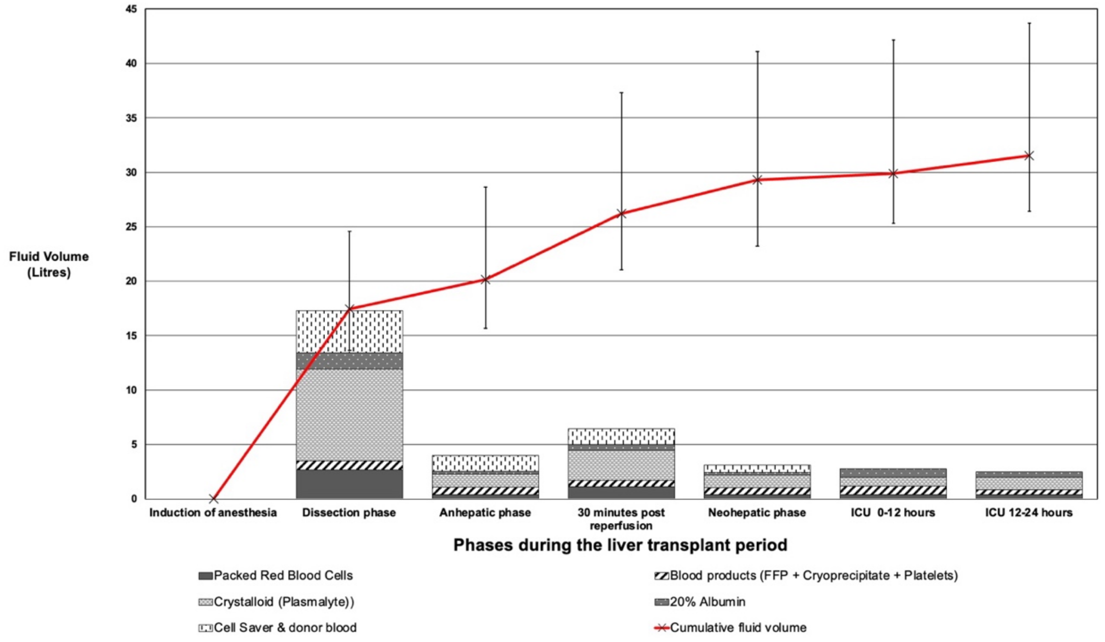

3. **Reviewer 1, comment 3:** While the study evaluated the relationship between FFP to PRBC ratio and platelet to PRBC ratio, it did not investigate the cryoprecipitate to PRBC ratio. Given the importance of cryoprecipitate in managing coagulopathy, this omission limits the comprehensiveness of the study's analysis.

Authors' response to R1 comment 3: Thank you for this insightful comment. We agree that investigating the cryoprecipitate (CPP) to packed red blood cell (pRBC) ratio is highly relevant in the context of massive transfusion during liver transplantation, given the critical role of cryoprecipitate in correcting coagulopathy.

In response, we have conducted a detailed analysis examining the relationship between the CPP:pRBC ratio and key clinical outcomes. As demonstrated by the linear regression results presented in the table below, a higher CPP ratio was significantly associated with a reduced duration of mechanical ventilation (coefficient = –99.42, 95% CI: –189.85 to –8.99, P = 0.039). Furthermore, the analysis revealed a significant interaction between CPP ratio and pRBC transfusion (coefficient = 10.89, 95% CI: 5.1 to 16.69, P = 0.001), indicating that the beneficial effect of a higher CPP ratio on reducing ventilation time diminishes as the volume of pRBC transfusion increases.

Regarding length of hospital stay, the CPP ratio alone was not significantly associated with length of hospital stay (P = 0.576). However, the interaction term between pRBC and CPP ratio showed a significant positive association (coefficient = 0.89, 95% CI: 0.04 to 1.73, P = 0.047), suggesting that patients receiving both higher volumes of pRBC and higher CPP ratios experienced longer hospital stays. No significant associations were observed between the CPP:pRBC ratio and ICU length of stay.

These findings have been incorporated into the Results section and are detailed in Table 8 and an additional Supplementary File (Supp File 9).

It is important to note, as highlighted in the table below, that the estimated effects of the pRBC:CPP ratio on outcomes such as "return to theatre for bleeding" may be confounded by factors such as multicollinearity and the limited sample size relative to the incidence of these specific outcomes. This is evidenced by the extremely high (infinite) values of the estimates for "return to theatre for bleeding." For instance, CPP transfusion may be a consequence of severe intra- and postoperative bleeding, which could itself be associated with prolonged mechanical ventilation and extended hospital stay. Therefore, these specific findings should be interpreted with caution, and we recommend focusing on the significant trends rather than the absolute magnitude of the estimated effects.

We have also addressed these limitations in the relevant section of the manuscript.

	**PRBC**		**CPP ratio**		**Interaction**	
	**Coefficient**	**P value**	**Coefficient**	**P value**	**Coefficient**	**P value**
Mechanical ventilation support (hours)	-2.7 (-15.06 – 9.66)	0.671	-99.42 (-189.85 – -8.99)	0.039*	10.89 (5.1 – 16.69)	0.001*
Length of hospital stay	0.79 (-0.99 – 2.57)	0.39	-3.82 (-17.1 – 9.46)	0.576	0.89 (0.04 – 1.73)	0.047*
Length of ICU stay	0.35 (-0.77 – 1.47)	0.541	-3.33 (-11.52 – 4.86)	0.431	0.53 (0.01 – 1.06)	0.055
Presence of any liver transplantation specific complication†	0.76 (0.53 – 1.08)	0.125	0.05 (0 – 1.82)	0.101	1.13 (0.94 – 1.36)	0.185
Liver transplantation-specific complication: bleeding†	21.88 (0 – inf)	>0.99	3131021.92 (0 – inf)	>0.99	0.43 (0 – inf)	>0.99
Liver transplantation-specific complication: Graft non-function†	2.36 (0 – inf)	>0.99	0 (0 – inf)	>0.99	1.66 (0 – inf)	>0.99

Liver transplantation-specific complication: Long-term failure†	1 (0 – inf)	>0.99	1 (0 – inf)	>0.99	1 (0 – inf)	>0.99
Liver transplantation-specific complication: Bile leakage†	1.2 (0 – inf)	>0.99	6723.47 (0 – inf)	>0.99	1.61 (0 – inf)	>0.99
Liver transplantation-specific complication: Hepatic artery/vein thrombosis†	0.01 (0 – inf)	>0.99	0 (0 – inf)	>0.99	4.47 (0 – inf)	>0.99
Liver transplantation-specific complication: Liver abscess†	0.74 (0 – inf)	>0.99	0.06 (0 – inf)	>0.99	1.23 (0 – inf)	>0.99
Liver transplantation specific complication: Others†	0 (0 – inf)	>0.99	0 (0 – inf)	>0.99	4015931.97 (0 – inf)	>0.99
Presence of any complication†	0.14 (0 – inf)	>0.99	0 (0 – inf)	>0.99	2.49 (0 – inf)	>0.99
No. of complications (≥3) †	1.21 (0.88 – 1.67)	0.229	4.12 (0.45 – 37.29)	0.208	0.93 (0.79 – 1.09)	0.36
Severe complications (CVD ≥ 3) †	1.13 (0.71 – 1.78)	0.609	0.28 (0 – 20.67)	0.559	1.08 (0.87 – 1.35)	0.469
Returned to the theatre: All-cause†	11017.17 (0 – inf)	>0.99	89534512283.13 (0 – inf)	>0.99	0.15 (0 – inf)	>0.99
Returned to the theatre: Bleeding†	$e^{1.12*10^{13}}$ (Inf – Inf)	<0.001*	$e^{4.11*10^{14}}$ (Inf – Inf)	<0.001*	$e^{-4.69*10^{13}}$ (0 – 0)	<0.001*
Returned to the theatre: Infection†	1 (0 – inf)	>0.99	1 (0 – inf)	>0.99	1 (0 – inf)	>0.99
Returned to the theatre: Infection†	3683.76 (0 – inf)	>0.99	1678967269852.81 (0 – inf)	>0.99	0.19 (0 – inf)	>0.99
On-table mortality†	10.48 (0 – inf)	>0.99	54122470.02 (0 – inf)	>0.99	0.27 (0 – inf)	>0.99
In-hospital mortality†	1 (0 – inf)	>0.99	1 (0 – inf)	>0.99	1 (0 – inf)	>0.99
30-day mortality†	8.39 (0 – inf)	>0.99	186.39 (0 – inf)	>0.99	0.5 (0 – inf)	>0.99
1-year mortality†	47.73 (0 – inf)	>0.99	1156.01 (0 – inf)	>0.99	0.32 (0 – inf)	>0.99
5-year mortality†	18577653.65 (0 – inf)	>0.99	7.31054729355929E+20 (0 – inf)	>0.99	0.07 (0 – inf)	>0.99
Overall mortality†	413457.5 (0 – inf)	>0.99	130403.66 (0 – inf)	>0.99	0.89 (0 – inf)	>0.99

4. **Reviewer 1, comment 4:** The use of antifibrinolytic agents such as tranexamic acid or amicar was not explored. These medications are crucial in managing bleeding and could significantly impact transfusion requirements and clinical outcomes.

Authors' response to R1 comment 4: Thank you for this important comment.

We have reviewed each medical record. In total 81 patients (1005) received tranexamic acid (2 grams) during the dissection or anhepatic phase. Five patients (61.7%) had evidence of hyperfibrinolysis on point-of-care TEG during the neohepatic phase and each received an additional 2 grams of tranexamic acid. This information has now been incorporated into the Results section.

We did not perform statistical analysis on the use of tranexamic acid, as all patients received at least one dose and only a small subset (n = 5) received an additional dose. The limited variability in tranexamic acid administration precludes meaningful statistical analysis regarding its impact on bleeding, fluid, or blood product requirements due to insufficient statistical power.

Reviewer 1, comment 5: The study did not address the potential cardiac effects of ultra-massive transfusion, especially right heart failure.

Authors' response to R1 comment 5: Thank you for this very important and insightful comment.

At our centre, transesophageal echocardiography (TEE) is increasingly employed during adult liver transplantation (LT), particularly in the context of massive transfusion, due to its capacity to provide real-time, dynamic assessment of cardiac function, volume status, and intraoperative complications. Our practice is consistent with recommendations from multiple national and international anaesthesiology societies, as well as recent systematic reviews, which advocate for TEE monitoring during LT to facilitate dynamic cardiac assessment and guide clinical interventions.

Continuous intraoperative visualization of cardiac chambers and function via TEE enables the immediate identification of the etiology of hemodynamic instability, such as hypovolemia, right ventricular (RV) failure, cardiac tamponade, or embolic phenomena. In our experience, TEE offers critical guidance for the administration of fluids and blood products, optimizing volume status and mitigating the risks of both hypovolemia and fluid overload—the latter of which may precipitate pulmonary edema and RV failure. Moreover, TEE facilitates the rapid diagnosis of intraoperative complications, including pulmonary embolism, intracardiac thrombus, pericardial effusion, and air embolism, thereby enabling prompt and targeted intervention.

In our practice for liver transplantation, TEE is utilized for both qualitative and quantitative assessments of right and left ventricular function. Quantitative indices such as tricuspid annular plane systolic excursion (TAPSE) less than 1.6 cm and RV fractional area change (RVFAC) below 35% are employed to identify RV dysfunction. We favor TAPSE for its ease of measurement and reproducibility, while RVFAC correlates strongly with RV ejection fraction and is sensitive to changes in RV systolic performance. During episodes of massive transfusion, visual assessment of RV size (dilatation and hypokinesis indicating RV overload or failure), right atrial enlargement (suggestive of RV dysfunction), and abnormal septal motion (paradoxical or flattened motion indicative of RV pressure overload) provides immediate evidence of RV dysfunction or acute reperfusion events.

In our recent case series, intraoperative TEE was performed in 65 patients (80%). TEE was withheld in 16 patients (12%), primarily due to the presence of grade 3 or actively bleeding esophageal varices (n = 12), esophageal strictures (n = 2), esophageal diverticulum (n = 1), or recent esophageal surgery (n = 1). Intraoperative evidence of RV dysfunction was identified in six patients; of these, one patient died intraoperatively, while RV function normalized postoperatively in the remaining five patients.

These findings have been incorporated into the manuscript. We anticipate that the inclusion of these results will enhance understanding of the interplay between massive transfusion and perioperative cardiac dysfunction in liver transplantation.

References

1. De Marchi L, Wang CJ, Skubas NJ, et al. Safety and Benefit of Transesophageal Echocardiography in Liver Transplant Surgery: A Position Paper From the Society for the Advancement of Transplant Anesthesia (SATA). Liver Transpl. 2020 Aug;26(8):1019-1029. doi: 10.1002/lt.25800. PMID: 32427417.
2. Hansebout C, Desai TV, Dhir A. Utility of Transesophageal Echocardiography During Orthotopic Liver Transplantation: A Narrative Review. Annals of Cardiac Anaesthesia 26(4):p 367-379, Oct–Dec 2023. | DOI: 10.4103/aca.aca_186_2
3. Gouvêa G, Feiner J, Joshi S, et al. (2022) Evaluation of right ventricular function during liver transplantation with transesophageal echocardiography. PLoS ONE 17(10): e0275301. https://doi.org/10.1371/journal.pone.0275301 [8][10].

4. De Pietri L, Mocchegiani F, Leuzzi C, et al. Transoesophageal echocardiography during liver transplantation. World J Hepatol 2015; 7(23):24322448 [PMID:26483865DOI:10.4254/wjh.v7.i23.2432]

5. **Reviewer 1, comment 6:** The precision of the reported data in Table 2 is excessive. For example, a total fluid volume reported as 31917.7 mL implies a level of precision that is not practical given the inherent variability and error in clinical measurements.

Authors' response to R1 comment 6: Thank you for this excellent suggestion. We agree that the precision reported in some of the tables is excessive and implies a level of precision that is not practical and is prone to error in clinical measurement. These have all been converted to Litres. This has been corrected throughout the manuscript, inclusive of all Tables.

6. **Reviewer 1, comment 7:** Similarly, the total norepinephrine use in Table 3 should be presented with appropriate rounding to reflect real-world clinical practice more realistically.

Authors' response to R1 comment 7: Thank you again for this suggestion, as above, we have adjusted **Table 3** accordingly.

Once again, our most sincere thanks to the Editor for dedicating their valuable time and expertise to review our manuscript. We deeply appreciate your thoughtful feedback and constructive suggestions, which have greatly contributed to improving the clarity and quality of our work. Your guidance has been instrumental in helping us refine our review, and we are grateful for the opportunity to benefit from your insights.

We hope that our manuscript is now suitable for publication in PLoS One. We would welcome the opportunity to make further modifications if needed.

Sincerely,

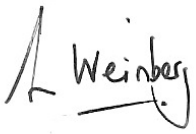

Professor Laurence Weinberg
BSc, MBBCh, MRCP, DipCritCareEcho, FANZCA, MD, PhD, ExecDipOL(Oxford)

Director, Department of Anaesthesia, Austin Hospital
Professor, Department of Critical Care, University of Melbourne, Australia
