# Supplementary material for: Ultra-massive fluid transfusion in adult liver transplant recipients: A single center observational study
Source: PLoS One. 2025 Jun 17;20(6):e0325829. doi: 10.1371/journal.pone.0325829 (PMC12173374; doi:10.1371/journal.pone.0325829)
Supplement: Attachment — Submitted filename: Response to Reviewers_8May2025.pdf [file pone.0325829.s014.pdf]

Ryan M. Thomas, M.D
Academic EditorPLoS ONE

Dear Dr. Thomas

Ref: Resubmission of PONE-D-25-06585R1

Title: Ultra-massive fluid transfusion in adult liver transplant recipients: a single center observational study

Once again, my coauthors and I would like to thank you and the expert reviewers for taking the time to provide a very constructive and thoughtful review of our manuscript.

We deeply appreciate your thoughtful feedback and constructive suggestions, which have contributed to improving the clarity and quality of our work. We are pleased that there are now minor revisions that have been requested. Accordingly, we have provided a detailed response to the important points raised by Reviewer 1.

As requested, we have included the following items with our resubmission:

1. A point-by-point response letter (to follow) that addresses each issue raised by the Academic Editor and the Reviewers. I have uploaded this letter as a separate file labelled "Response to Reviewers."
2. A marked-up copy of the manuscript that highlights changes made to the original version. This has been uploaded as a separate file labelled "Revised Manuscript with Tracked Changes."
3. An unmarked version of our revised paper without tracked changes. This has also been uploaded as a separate file labelled "Manuscript."

Comments by Reviewer 1

1. **Reviewer 1, comment:** The authors have performed much of the additional data collection and analyses requested. I also accept their rationale for not performing an analysis of the predictors of UMFT. There are two subanalyses from the newly revealed data that would beneficial to the manuscript.

We sincerely appreciate your expert insights, and the time dedicated to reviewing our manuscript. As a result of your thoughtful feedback, the manuscript has been significantly strengthened and improved. We are grateful for the opportunity to address the two questions raised below.

Reviewer 1, Question 1: The authors have done the work to parse the time period during which the fluids were given to the patients. Was there any specific period of time when volume was administered that was most correlated with outcomes? Or was there only a significant correlation when looking at total volume? It would be important to know whether intraop versus postop fluid was most correlated, or perhaps even prehepatectomy versus post reperfusion.

Authors' response to Question 1: Thank you for this every insightful question. We have now undertaken further analysis investigating the associations between intraoperative and postoperative fluid/transfusion volumes and postoperative recovery outcomes. In addition, we have further the evaluated the associations between intraoperative and postoperative fluid/transfusion volumes and postoperative complications. These additional analyses have been included in the revised manuscript.

As seen in Table 1 below, we have used the term "resuscitation shift", which we have operationally defined as the net change in postoperative fluid or transfusion volume administered compared to the volume of fluid administration intraoperatively. Two regression models were adjusted for perioperative

covariates to analyze these relationships. Models for intraoperative and postoperative variables of fluid or transfusion were fitted simultaneously to assess their independent associations with clinical outcomes. As a supplementary sensitivity analysis, the resuscitation shift model (which we have termed the "trajectory model") quantified the net volume difference between the intraoperative and postoperative 24-hour periods.

Our findings show that intraoperative and postoperative administration of fluids, packed red blood cell (RBC) units, and other blood products demonstrated statistically significant associations with adverse postoperative recovery metrics, including duration of mechanical ventilation and length of stay in both intensive care units (ICU) and hospital settings (**See Table 1 below**). The magnitude of association was more pronounced for postoperative fluid volumes, packed RBC units, and transfusions compared to their intraoperative counterparts, as evidenced by larger regression coefficients. The negative coefficients observed for resuscitation shift parameters suggest that increased postoperative fluid administration and transfusion (represented by smaller shift values) correlated with poorer clinical outcomes, thus highlighting the potential deleterious effects of excessive fluid or blood product administration during the early postoperative period.

These statistically significant findings warrant cautious interpretation. While elevated fluid or transfusion volumes may contribute to adverse outcomes, reverse causality cannot be definitively excluded from consideration. Patients experiencing prolonged ICU or hospital stays due to complications unrelated to hemorrhage (such as sepsis or organ dysfunction) may have received additional fluids or blood products as part of their supportive care regimen. This bidirectional relationship inherently complicates causal inference in statistical regression models. Although we implemented trajectory modeling as a sensitivity analysis, this approach also presents methodological limitations. The resuscitation shift parameter indicates that fluid administration or transfusion at different perioperative phases represents distinct physiological responses that reflect variations in bleeding patterns, hemodynamic stability, and resuscitation requirements. However, identical resuscitation shift values (e.g., resuscitation shift = 0) may represent fundamentally different clinical scenarios-for instance, minimal intraoperative plus minimal postoperative volumes versus substantial intraoperative plus substantial postoperative volumes. The former scenario would reflect outcomes associated with conservative resuscitation strategies, whereas the latter would demonstrate outcomes related to aggressive volume replacement approaches.

Table 2 below shows our analysis of discrete fluid volumes and transfusion quantities administered during intraoperative or postoperative 24-hour periods revealed that packed red blood cell (RBC) unit counts demonstrated a statistically significant association with both surgery-specific complications and severe postoperative complications (Clavien-Dindo Classification [CDC] grade ≥ 3). Postoperative RBC transfusion was associated with a lower risk of surgery-specific complications (odds ratio [OR] = 0.35, 95% confidence interval [CI]: 0.13–0.97, $p = 0.043$), potentially reflecting therapeutic benefits from timely correction of postoperative anemia or active hemorrhage. However, only 15.8% of surgery-specific complications in this cohort were attributable to bleeding, necessitating further granular data to clarify the role of postoperative transfusion in mitigating such outcomes.

Table 1. Associations between intraoperative and postoperative fluid/transfusion volumes and postoperative recovery outcomes, including resuscitation shift.

**Postoperative complication**	**Variable**	**Time point**	**β (95%CI)**	**P value**
Mechanical ventilation (hours)	Total volume (mL)	Intraoperative	4.0 (0.7 – 7.3)	0.021*
		Postoperative 24hr	44.2 (21.2 – 67.2)	<0.001*
		Resuscitation shift	–5.3 (–9.3 – –1.2)	0.013*
	RBC transfusion (units)	Intraoperative	9.20 (1.73 – 16.67)	0.017*
		Postoperative 24hr	43.89 (28.03 – 59.75)	<0.001*
		Resuscitation shift	–2.38 (–13.01 – 8.24)	0.651
	Transfusion volume (100mL)	Intraoperative	21 (7 –34)	0.004*
		Postoperative 24hr	67 (45 – 89)	<0.001*
		Resuscitation shift	–7 (–27 – 14)	0.532
Hospital LOS (days)	Total volume (mL)	Intraoperative	0.73 (0.27 – 1.19)	0.003*
		Postoperative 24hr	3.31 (0.11 – 6.50)	0.044*
		Resuscitation shift	-0.75 (-1.24 – -0.27)	0.004*
	RBC transfusion (units)	Intraoperative	1.87 (0.74 – 3.00)	0.002*
		Postoperative 24hr	3.83 (1.41 – 6.25)	0.003*
		Resuscitation shift	–0.98 (–2.25 – 0.30)	0.132
	Transfusion volume (100mL)	Intraoperative	4.48 (2.30 – 6.66)	<0.001*
		Postoperative 24hr	4.75 (1.26 – 8.24)	0.011*
		Resuscitation shift	-2.68 (-5.24 – -0.12)	0.041*
ICU LOS (days)	Total volume (mL)	Intraoperative	0.32 (0.05 – 0.60)	0.021*
		Postoperative 24hr	3.25 (1.40 – 5.10)	0.001*
		Resuscitation shift	–0.42 (–0.74 – –0.10)	0.012*
	RBC transfusion (units)	Intraoperative	0.78 (0.14 – 1.43)	0.019*
		Postoperative 24hr	3.04 (1.66 – 4.42)	<0.001*
		Resuscitation shift	-0.29 (–1.12 – 0.54)	0.483

	Transfusion volume (100mL)	Intraoperative	2.00 (0.76 – 3.23)	0.002*
		Postoperative 24hr	4.15 (2.06 – 6.24)	<0.001*
		Resuscitation shift	–1.00 (–2.68 – 0.68)	0.235

The term resuscitation shift was operationally defined as the net change in postoperative fluid or transfusion volume administered compared to the volume of fluid administration intraoperatively. Two regression models were adjusted for perioperative covariates to analyze these relationships. Models for intraoperative and postoperative variables of fluid or transfusion were fitted simultaneously to assess their independent associations with clinical outcomes. As a supplementary sensitivity analysis, the resuscitation shift model (termed the trajectory model) quantified the net volume difference between the intraoperative and postoperative 24-hour periods. *: $P < 0.05$.

Table 2. Associations between intraoperative and postoperative fluid/transfusion volumes and postoperative complications, including resuscitation shift.

**Postoperative complication**	**Variable**	**Time point**	**OR (95%CI)**	**P value**
Surgery-specific complications	Total volume (mL)	Intraoperative	0.98 (0.92 – 1.03)	0.574
		Postoperative 24hr	0.60 (0.31 – 1.14)	0.125
		Resuscitation shift	1.02 (0.96 – 1.08)	0.448
	RBC transfusion (units)	Intraoperative	1.13 (0.91 – 1.41)	0.268
		Postoperative 24hr	0.35 (0.13 – 0.97)	0.043*
		Resuscitation shift	0.95 (0.83 – 1.09)	0.460
	Transfusion volume (100mL)	Intraoperative	100.0 (99.97 – 100.06)	0.735
		Postoperative 24hr	99.91 (99.80 – 100.01)	0.106
		Resuscitation shift	99.99 (99.97 – 100.01)	0.589
Surgery specific complications: Bleeding	Total volume (mL)	Intraoperative	-	-
		Postoperative 24hr	-	-
		Resuscitation shift	-	-
	RBC transfusion (units)	Intraoperative	-	-
		Postoperative 24hr	-	-
		Resuscitation shift	-	-

	Transfusion volume (100mL)	Intraoperative	-	-
		Postoperative 24hr	-	-
		Resuscitation shift	-	-
No. of complications ≥ 3	Total volume (mL)	Intraoperative	1.01 (0.96 – 1.06)	0.801
		Postoperative 24hr	1.00 (0.63 – 1.57)	0.986
		Resuscitation shift	0.99 (0.94 – 1.05)	0.802
	RBC transfusion (units)	Intraoperative	0.93 (0.60 – 1.43)	0.726
		Postoperative 24hr	1.07 (0.91 – 1.26)	0.372
		Resuscitation shift	0.94 (0.83 – 1.07)	0.348
	Transfusion volume (100mL)	Intraoperative	100.01 (99.98 – 100.04)	0.406
		Postoperative 24hr	100.00 (99.94 – 100.06)	0.997
		Resuscitation shift	99.99 (99.97 – 100.02)	0.450
CDC grade ≥ 3	Total volume (mL)	Intraoperative	1.01 (0.95 – 1.07)	0.659
		Postoperative 24hr	1.49 (0.96 – 2.33)	0.074
		Resuscitation shift	0.99 (0.94 – 1.05)	0.748
	RBC transfusion (units)	Intraoperative	1.25 (0.93 – 1.70)	0.126
		Postoperative 24hr	1.49 (1.00-2.22)	0.048*
		Resuscitation shift	0.92 (0.80 – 1.06)	0.298
	Transfusion volume (100mL)	Intraoperative	100.02 (99.98 – 100.06)	0.2142
		Postoperative 24hr	100.05 (99.99 – 100.11)	0.088
		Resuscitation shift	99.99 (99.96 – 100.02)	0.514
Return to theatre: Bleeding	Total volume (mL)	Intraoperative	-	-
		Postoperative 24hr	-	-
		Resuscitation shift	-	-
	RBC transfusion (units)	Intraoperative	-	-
		Postoperative 24hr	-	-

		Resuscitation shift	-	-
	Transfusion volume (100mL)	Intraoperative	99.93 (99.85 – 100.02)	0.132
		Postoperative 24hr	100.07 (99.94 – 100.19)	0.292
		Resuscitation shift	100.07 (99.99 – 100.15)	0.119
30-day mortality	Total volume (mL)	Intraoperative	-	-
		Postoperative 24hr	-	-
		Resuscitation shift	-	-
	RBC transfusion (units)	Intraoperative	-	-
		Postoperative 24hr	-	-
		Resuscitation shift	-	-
	Transfusion volume (100mL)	Intraoperative	-	-
		Postoperative 24hr	-	-
		Resuscitation shift	-	-

The term resuscitation shift was operationally defined as the net change in postoperative fluid or transfusion volume administered compared to the volume of fluid administration intraoperatively. Two logistic regression models were adjusted for perioperative covariates to analyze these relationships. Models for intraoperative and postoperative variables of fluid or transfusion were fitted simultaneously to assess their independent effects. As a supplementary sensitivity analysis, the resuscitation shift model (termed the trajectory model) quantified the net volume difference between intraoperative and postoperative 24-hour periods. –: denotes model convergence failure or insufficient data for reliable estimation. These models were excluded from interpretation due to instability or low event rates. *: P<0.05.

While we thank the reviewer for this important comment, suboptimal model fit across multiple regression analyses warrants cautious interpretation. Potential contributors to model instability include limited sample size, imbalance between complicated and uncomplicated cases, and the absence of a control group for comparative assessment. Consequently, our granular analyses may be susceptible to bias, and inferences should prioritize the magnitude of associations between fluid/transfusion volumes and outcomes rather than precise effect estimates.

To enhance methodological transparency, we have addressed these limitations explicitly and would prefer relegating our detailed sensitivity analyses to supplementary materials contingent upon editorial request. We hope that our response to this question maintains analytical rigor while aligning with standards of academic discourse.

Reviewer 1, Question 2: Similarly, given the number of episodes of right heart failure they observed, were there correlations with intraoperative fluid administration? While there may be too few events to do proper statistics, some further detail would be helpful to understand the scenarios.

Authors' response to Question 2: Thank you for this question. We acknowledge the Reviewer's suggestion regarding the association between right cardiac dysfunction and intraoperative fluid administration. However, we must respectfully discuss that such an analysis is not statistically feasible with our current dataset. Specifically, intraoperative evidence of right ventricular dysfunction was identified in only six patients within our entire cohort, representing too few events to conduct meaningful statistical analysis or draw valid conclusions. Despite this limitation, we have performed a comparative assessment of these six patients with documented RV dysfunction against the remainder of the cohort, examining perioperative fluid volumes, use of blood products, and postoperative complications. As anticipated, this comparison revealed no statistically significant differences in any of these parameters between the groups. The paucity of events (n=6) substantially limits statistical power and increases the risk of both Type I and Type II errors, rendering any formal statistical inference unreliable.

While we appreciate the clinical relevance of this question, we believe that a properly powered study specifically designed to investigate this relationship would be required to generate meaningful conclusions about the association between right ventricular dysfunction and intraoperative fluid management strategies. We have noted this important limitation and potential avenue for future research in our discussion section.

Table 1: Comparison of fluid volumes and key outcomes for patients with intraoperative transesophageal features of intraoperative right cardiac dysfunction compared to patients without right sided cardiac dysfunction.

Variable	Patients with intraoperative evidence of right heart dysfunction (n = 6)	Patients with no evidence of intraoperative evidence of right heart dysfunction (n = 66)	P-value
**Baseline characteristics**			
Age (years)	51 (33 – 64)	56 (50 – 62)	0.444
Model end stage liver disease	19 (15 – 26)	21 (14 – 27)	0.823
BMI (kg/m^2)	29 (33 – 64)	28 (24 – 33)	0.863
**Fluid volumes**			
Intraoperative PRBC (L)	5.25 (2.97 – 7.96)	4.55 (3.15 – 5.69)	0.507
Intraoperative FFP (L)	1.5 (0.9 – 2.62)	1.5 (1.0 – 2.0)	>0.99
Intraoperative cryoprecipitate (L)	0.90 (0.6 – 1.20)	0.56 (0.35 – 0.70)	>0.99

Intraoperative platelets (L)	1.46 (0.8 – 1.87)	0.98 (0.65 – 1.30)	0.091
Intraoperative albumin (L)	1.8 (1.2 – 2.35)	2.55 (2.15 – 3.2)	0.016
Intraoperative plasmalyte (L)	10.0 (3.75 – 14.25)	14.0 (11.0 – 18.0)	0.059
Total intraoperative volumes (L)	31.32 (27.46 – 70.34)	33.31 (28.53 – 46.25)	0.945
Total ICU volumes (L)	0.86 (0.22 – 5.23)	3.4 (2.63 – 4.60)	0.108
Total intraoperative and 24-hour postoperative volume (L)	31.52 (29.97 – 72.16)	37.19 (31.70 – 48.73)	0.684
**Key outcomes**			
ICU length of stay (days)	5.0 (4.0 – 20.0)	5 (3.0 – 14)	0.661
Total hospital length of stay (days)	28.0 (14.0 – 44.0)	19 (14.0 – 38.0)	0.773
On table mortality (number of patients)	1 (16.7%)	0 (0%)	0.083
Mortality at 30 days (number of patients)	1 (16.7%)	4 (60.6%)	0.361

Once again, our most sincere thanks to the Editor and Reviewer 1 for dedicating their valuable time and expertise to review our manuscript. We deeply appreciate your thoughtful feedback and constructive suggestions, which have greatly contributed to improving the clarity and quality of our work. Your guidance has been instrumental in helping us refine our review, and we are grateful for the opportunity to benefit from your insights.

We hope that our manuscript is now suitable for publication in PLoS One. We would welcome the opportunity to make further modifications if needed.

Sincerely,

Professor Laurence Weinberg
BSc, MBBCh, MRCP, DipCritCareEcho, FANZCA, MD, PhD, ExecDipOL(Oxford)

Director, Department of Anaesthesia, Austin Hospital
Professor, Department of Critical Care, University of Melbourne, Australia
